# Outcomes of bariatric surgery in the setting of compensated advanced chronic liver disease associated with clinically significant portal hypertension: a multicenter, retrospective, cohort study on feasibility and safety

**DOI:** 10.1097/JS9.0000000000001310

**Published:** 2024-03-18

**Authors:** Victor Temime, Omar M. Ghanem, Julie K. Heimbach, Tayyab S. Diwan, Hadrien Tranchart, Hussein Abdallah, Claire Blanchard, Marie Lontrichard, Fabian Reche, Anne-Laure Borel, Amanda Belluzzi, Mirto Foletto, Emilio Manno, Tigran Poghosyan, Andrea Chierici, Antonio Iannelli

**Affiliations:** a Centre Hospitalier Universitaire de Nice-Digestive Surgery and Liver Transplantation Unit, Archet 2 Hospital, Nice; b Université Paris Cité, AP-HP.Nord, Hôpital Bichat Claude Bernard, Service de Chirurgie Digestive UMR 1149, Inserm, Paris; c Université Côte d’Azur, Nice; d Department of Minimally Invasive Digestive Surgery, Antoine Béclère Hospital, AP-HP, Clamart; Paris-Saclay University, Orsay; e Clinique de chirurgie cancérologique, digestive et endocrinienne, institut des maladies de l’appareil digestif (IMAD), CHU de Nantes; CHU de Nantes, l’institut du thorax, Nantes université, CNRS, Inserm, Nantes; f Univesity Grenoble Alpes, Department of Digestive Surgery; g Department of Endocrinology Diabetology Nutrition, Grenoble Alpes University Hospital, Centre Spécialisé de l’Obésité Grenoble Arc Alpin, Grenoble, France; h Bariatric Surgery Unit, University of Padua, Padua; i AORN A. Cardarelli Napoli, UO Chirurgia Bariatrica e Metabolica, Napoli, Italy; j Department of Surgery; k Division of Transplantation, Department of Surgery, Mayo Clinic, Rochester, Minnesota, USA; l Inserm, U1065, Team 8 ‘Hepatic complications of obesity and alcohol’; m ADIPOCIBLE Study Group

**Keywords:** bariatric surgery, bleeding, liver cirrhosis, liver transplantation, mortality, portal hypertension, portal thrombosis

## Abstract

**Background:**

The obesity epidemic has led to an increase in the proportion of patients with chronic liver disease due to metabolic associated steatosic liver disease and in the prevalence of obesity in patients with cirrhosis. Metabolic and bariatric surgery (MBS) has been proven to determine weight loss, obesity-related medical problems remission, and liver steatosis, inflammation, and fibrosis improvement. However, cirrhosis and portal hypertension are well-known risk factors for increased morbidity and mortality after surgery. The aim of this study is to evaluate the safety of MBS in patients with compensated advanced chronic liver disease (cALCD) and clinically significant portal hypertension (CSPH).

**Material and methods:**

This is an international, multicentric, retrospective study on 63 individuals affected by obesity with cALCD and CSPH who underwent MBS in tertiary referral centers with experts hepatobiliary surgeons between January 2010 and October 2022. The primary endpoint was postoperative mortality at 90 days. The secondary endpoints included postoperative weight loss at last follow-up and postoperative complication rate. In addition, the authors performed subgroup analyses of Child-Pugh (A vs. B) score, MELD (≤9 vs. >9) score, and type of surgery.

**Results:**

One patient (1.6%) experienced gastric leakage and mortality. There were three (5%) reported cases of portal vein thrombosis, two (3%) postoperative acute renal failure, and one (1.6%) postoperative encephalopathy. Child-Pugh score A resulted to be a protective factor for intraoperative bleeding requiring transfusion at univariate analysis (OR: 0.73, 95% CI: 0.55–0.97, *P*=0.046) but not at multivariate analysis. MELD>9 score and the type of surgery did not result to be a risk factor for any postoperative complication.

**Conclusion:**

MBS is safe in patients with cALCD and CSPH performed in tertiary bariatric referral centers with hepatobiliary expert surgeons. Larger, prospective studies with longer follow-up periods are needed to confirm these results.

## Introduction

Highlights

- Obesity affects 30% of patients with liver cirrhosis.
- Liver cirrhosis has been considered a contraindication for metabolic and bariatric surgery till recently.
- We showed that metabolic and bariatric surgery in patients with compensated liver cirrhosis has low morbidity (1.6%) and mortality (1.6%).
- Patients with Child-Pugh A score have lower risk (OR: 0.73) of intraoperative bleeding requiring blood transfusion compared to those with Child-Pugh B score.
- No difference in postoperative morbidity and mortality was found depending on the type of surgical procedure.

The global obesity epidemic has led to an increase in the proportion of patients with chronic liver disease due to metabolic associated steatosic liver disease and in the prevalence of obesity in patients with cirrhosis of all etiologies. The reported prevalence of obesity in patients with cirrhosis is 30%, which appears to be similar to that in the general population^1^.

Metabolic and bariatric surgery (MBS) is currently considered the most effective and durable treatment for morbid obesity, as it is associated with remission and/or improvement of many obesity-related comorbidities and improved quality and length of life^2^.

However, the surgical risk of MBS is increased in individuals with liver cirrhosis compared to those without cirrhosis, and determining the risk-benefit ratio of surgery in this setting is a complex task, further complicated by the lack of randomized controlled trials^3^. Mosko and Nguyen^4^ reported a threefold increased mortality rate for MBS in the setting of compensated advanced chronic liver disease (cACLD) compared with those without (0.9 vs 0.3%) in United States Nationwide Inpatient Sample study between 1998 and 2007.

Interestingly, the authors also showed that mortality was dramatically higher in case of decompensated cirrhosis (16.3%) that was clearly identified as a contraindication to MBS^4^. However, this study was published more than 10 years ago, and the mortality of MBS has decreased significantly and is currently around 0.1%^5^. Furthermore, with the introduction of transient elastography into clinical practice patients with chronic liver disease can be easily identified before surgery avoiding the incidental diagnosis of cirrhosis during surgery and favoring the referral of these cases to tertiary referral high-volume centers. Perioperative management of patients with chronic liver disease impose liver dysfunction, portal hypertension, and cardiopulmonary and renal comorbidity precise assessment in order to predict postoperative morbidity and mortality, which also depends on the complexity and the type of surgery^6^.

Among individuals with morbid obesity and cACLD some may also have clinically significant portal hypertension (CSPH) representing a subset of patients that deserve particular attention. The concept of CSPH defined in Baveno VI plays a major role in Baveno VII, and is defined by the presence of a porto-caval gradient ≥10 mmHg using an inflatable balloon catheter without anesthesia. As in current clinical practice it is rarely possible to do this except in expert centers, the diagnosis of CSPH is therefore based on the presence of ascites, including ascites that is only radiological, or the presence of oeso-gastric varices of any size, with or without a red sign and regardless of the Child-Pugh score, or the presence of porto-systemic shunts (e.g. repermeabilised paraumbilical vein, spleno-renal shunt, etc.), or the measurement of an elasticity ≥25 kPa. Splenomegaly alone is not considered to be a sign of CSPH^7^.

While a CSPH is currently considered as a contraindication to MBS by most^3^, a few series with limited numbers of patients have been published suggesting that CSPH should not be considered a formal contraindication to MBS^8–10^. However, evidence of the feasibility and safeness of MBS in the setting CSPH is scarce and comes from monocentric series and a single metanalysis including 32 cases^11^.

This study aims to evaluate the outcomes of MBS in individuals with morbid obesity and cACLD associated with CSPH in a large multicenter, multinational series in order to define its feasibility and safety.

## Material and methods

### Study design

This is a retrospective, multicenter, international study to investigate the feasibility and safety of MBS in the setting of CSPH. The trial was retrospectively registered on ClinicalTrials.gov under the name of BARIAPORTAL (ClinicalTrials.gov identifier: NCT05653115).

Only large tertiary referral centers for both MBS and liver transplantation were selected to participate in the study in order to select only patients that had received advanced care for both obesity and portal hypertension.

An e-mail was sent to major centers in Europe and United States to invite bariatric surgeons to participate in the study including individuals with morbid obesity and CSPH undergoing MBS from January 2010 to October 2022 with the aim of gathering in a single, multicentric, international series more than 50 cases (almost double of the cases published in the literature so far) with homogeneous inclusion criteria. Anonymized data were included in an Excel file.

The work has been reported in line with the strengthening the reporting of cohort, cross-sectional, and case–control studies in surgery (STROCSS) criteria^12^.

### Inclusion criteria

Individuals with morbid obesity (BMI ≥40 or ≥35 with at least one comorbidity among blood hypertension, type 2 diabetes, invalidating arthritis and sleep apnea syndrome) and CSPH (defined as HVPG ≥10 mmHg and/or cross-sectional imaging showing collateral circulation and/or varices on esophagogastroduodenoscopy) undergoing bariatric surgery.
Case selection by a multidisciplinary team including a liver surgeon and a hepatologist in the setting of a liver transplantation program.
Information available to determine postoperative mortality (at least the first postoperative month or any duration for primary hospitalization longer than 1 months).

### Exclusion criteria

Child-Pugh score C.
Lack of preoperative evidence of CSPH despite evidence of cACLD.
Patients with portal hypertension resolution after transjugular porto-systemic shunt (TIPS) positioning.
Lack of information to determine at least postoperative mortality.
Any other procedure synchronous with MBS.

### Outcomes

#### Primary outcome

Postoperative mortality defined as death occurring within 30 days from MBS or in the same hospitalization of index surgery (primary cause of death and if liver-related, postoperative day).

#### Secondary outcomes

General (age and sex), anthropometric measures [weight (kg), height (meters), BMI] and obesity linked comorbidities [blood hypertension (HT), sleep apnea syndrome (SAOS), type 2 diabetes (T2D)].
Etiology of liver disease [alcohol, viral (hepatitis B, C), NASH, other and use and timing of liver biopsy (preoperative vs during surgery)].
Symptoms and signs of past and postoperative liver decompensation (ascites and encephalopathy).
Preoperative work-up to define CSPH (use of transjugular pressure measurement (HVPG) and/or cross-sectional imaging and/or upper GI endoscopy to define CSPH)^7^.
Liver function (Child’s score, MELD score).
Strategy to reduce portal hypertension (use of TIPS; beta-blockers).
Type of MBS (gastric banding, sleeve gastrectomy, Roux-en-Y gastric bypass, or other).
Functional outcomes (% total body weight loss in kg).
Postoperative mortality and complications rate after stratification of the population based on Child-Pugh score, MELD score, and type of MBS.

### Statistical analysis

Patients’ characteristics were reported as numbers and percentages for categorical variables and means±SD or medians and interquartile ranges (IQR) depending on weather the distribution was normal or not. Continuous variables’ distribution was evaluated with the Shapiro–Wilk test.

Groups comparison was initially performed through the *χ*^2^ test, the *t*-Student test, and the Mann–Whitney *U* test. When *P* resulted to be ≤0.2, variables were selected to be included in a logistic regression model for univariate analysis to estimate odds ratios (OR) with their respective 95% CI. Multivariate analysis through stepwise backward logistic regression based on the Akaike Information Criterion (AIC) was then performed when univariate analysis resulted to be significant for a specific postoperative outcome, adjusted on between groups differences in baseline characteristics.

Statistical analysis was performed with R 4.1.2^13^.

## Results

**Figure 1:**
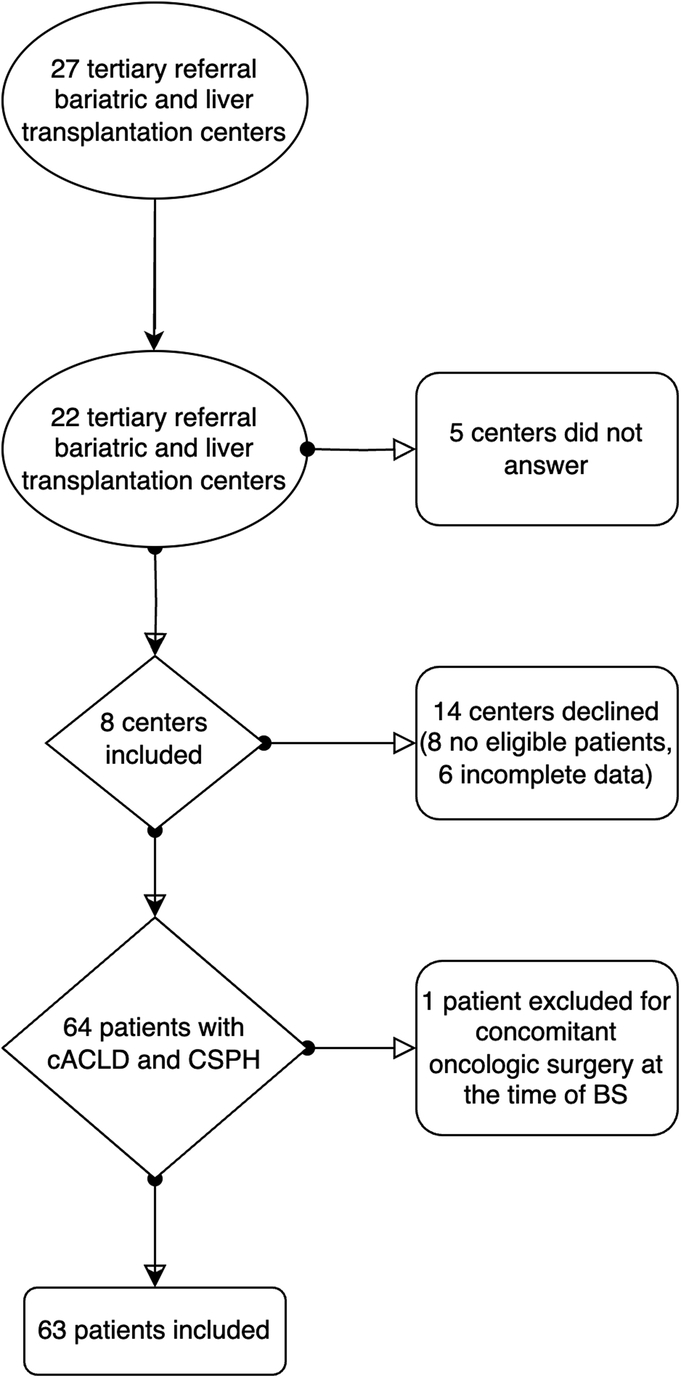
Flow-chart depicting patients inclusion process.

Between January 2010 and October 2022, 64 patients undergoing MBS with CSPH were included in the BARAPORTAL study. After revision of all the medical files received from the different centers, one patient was excluded as he underwent liver resection and right nephrectomy for hepatocellular hepatocarcinoma at the moment of MBS, introducing a bias for the evaluation of postoperative outcomes. Thus, 63 patients were finally included for analysis (Fig. 1).

### Characteristics of the study population

In the study population, median age was 56 (48–61) years, median preoperative weight was 124 (110–143) kg, and median preoperative BMI was 44.1 (38.9–51.2) kg/m^2^.

Regarding comorbidities, 40 (63%) patients had a history of cardiovascular disease, 46 (73%) had T2D, 44 (70%) had OSAS, 16 (25%) had CKD, and 3 (5%) had a history of DVT. In addition, 5 (8%) patients had ascites at the time of surgery, 25 (40%) had grade 1 EV, 7 (11%) had grade 2 EV, and 7 (11%) had undergone endoscopic EV ligation before surgery. The main cause of cirrhosis was NASH, which concerned 51 (81%) patients while 6 (10%) patients had viral cirrhosis (HBV-HCV). Another cause of chronic liver disease was found in 10 (16%) patients. Above all, five (8%) patients have multiples factors contributing to liver cirrhosis.

Liver disease was diagnosed on preoperative biopsy in 19 (30%) patients and on intraoperative biopsy in 44 (70%) patients. Preoperative TIPS was performed in five (8%) patients. Thirty-five (56%) patients had a MELD score between 6 and 9, 9 (14%) between 10 and 11, 7 (11%) between 12 and 15, and 9 (14%)≥16. Fifty-six (89%) patients had a Child-Pugh score A and 7 (11%) had a Child-Pugh score B. To identify CSPH, measurement of the HVPG was performed in one patient (2%), CT-scan in 31 (49%) patients and endoscopy in 49 (78%) patients.

The most frequently performed MBS was SG, concerning 47 (75%) patients. RYGB was done in 14 (22%) cases, and 2 (3%) of patients had GB.

Baseline characteristics of the included patients are resumed in Table 1.

**Table 1:** Baseline characteristics of the included patients.

	Study population (*n*=63)
Age, years	56 (48–61)
Sex, Male	26 (41)
Preoperative weight, kg	124 (110–143)
Preoperative BMI, kg/m2	44.1 (38.9–51.2)
Cardiovascular disease, *n*	40 (63)
T2D, *n*	46 (73)
OSAS, *n*	44 (70)
GERD, *n*	23 (37)
CKD, *n*	16 (25)
DVT, *n*	3 (5)
EV, *n*	34 (54)
grade 1, *n*	25 (40)
grade 2, *n*	7 (11)
EV ligation, *n*	7 (11)
Ascites, *n*	5 (8)
Alcohol consumption, *n*	10 (16)
Splenomegaly, *n*	56 (89)
Cirrhosis etiology	
NASH, *n*	51 (81)
Viral, *n*	6 (10)
HBV active replication	1 (2)
HCV active replication	2 (3)
Other, *n*	10 (16)
Liver biopsy, *n*	42 (67)
Preoperative, *n*	19 (30)
Intraoperative, *n*	44 (70)
TIPS, *n*	5 (8)
Diagnosis of CSPH	
HVPG, *n*	1 (2)
CT-scan, n	31 (49)
GI endoscopy, *n*	49 (78)
MELD score	
6–9, *n*	35 (56)
10–11, *n*	9 (14)
12–15, *n*	7 (11)
≥16, *n*	9 (14)
Child-Pugh	
A, *n*	56 (89)
B, *n*	7 (11)
ASA score	
2, *n*	3 (5)
3, *n*	48 (76)
4, *n*	7 (11)
ß-blockers treatment, *n*	39 (62)
Antihypertensive treatment, *n*	40 (63)
Insulin treatment, *n*	30 (48)

Categorical variables are expressed as *n* (%).

Continuous variables are reported as median (IQR).

CKD, chronic kidney disease; CSPH, clinically significant portal hypertension; CT, computed tomography; DT2, type 2 diabetes; DVT, deep vein thrombosis; EV, esophageal varices; GERD, gastro-esophageal reflux disease; GI, gastrointestinal; HVPG, hepatic venous-portal gradient; NASH, nonalcoholic steatohepatitis; OSAS, obstructive sleep apnea syndrome; TIPS, transjugular intrahepatic portosystemic shunt.

### Postoperative outcomes

One patient (1.6%) died within 90 days after MBS. Postoperative outcomes are reported in Table 2.

**Table 2:** Postoperative outcomes.

	Study population (*n*=63)
Bariatric procedure	
SG, *n*	47 (75)
RYGB, *n*	14 (22)
Other, *n*	2 (3)
Follow-up, months	38±21
Decompensation	
Postoperative encephalopathy, *n*	1 (1.6)
General complications	
Postoperative renal failure, *n*	2 (3)
Dialysis, *n*	1 (1.6)
Postoperative pulmonary embolism, *n*	0 (0)
Portal vein thrombosis, *n*	3 (5)
Reintubation, *n*	0 (0)
Infection	
Sepsis, *n*	0 (0)
Postoperative pneumonia, *n*	0 (0)
Ascites infection, *n*	0 (0)
MBS complications	
Intraoperative bleeding^a^, *n*	5 (8)
Early surgical postoperative complication, *n*	1 (1.6)
Leak, *n*	1 (1.6)
Mortality, *n*	1 (1.6)
Outcomes	
Postoperative weight, kg	98 (84.5–110.1)
Postoperative BMI^b^, kg/m^2^	34.8 (30.8–40.7)

Categorical variables are expressed as *n* (%).

Continuous variables are reported as median (IQR).

a Intraoperative bleeding requiring blood transfusion.

b Postoperative BMI at last follow-up.

RYGB, Roux-en-Y gastric bypass; SG, sleeve gastrectomy.

The only patient who died postoperatively in this series was a 66-year-old woman with a Child-Pugh A6 and MELD 12 HBV-related cACLD with endoscopic diagnosis of CSPH. This patient had a history of multiple liver thermal ablation for hepatocellular carcinoma prior to SG and was included in liver transplantation waiting-list at the moment of MBS. The indication for MBS was BMI=40 kg/m^2^ and T2D. Postoperative course was complicated by a gastric leak. Early laparoscopic washing and drainage, associated with antibiotic treatment and nihil per OS, was performed but the patients developed septic shock and died during the MBS hospitalization.

Median postoperative weight at last follow-up after MBS was 98 (84.5–110.1) kg, while median post operative BMI was 34.8 (30.8–40.7) kg/m^2^.

Intraoperative bleeding necessitating blood transfusion was reported for five (8%) patients.

Postoperatively, two (3%) patients developed acute renal failure, of whom one (2%) needed dialysis. Three (5%) cases of portal vein thrombosis were reported, and one (2%) patient needed reoperation.

None of the included patients developed ascites postoperatively. During follow-up, 12 patients of this series died: 1 for HCC evolution, 10 for cardiovascular disease, and 1 for COVID-related respiratory insufficiency.

### Child-Pugh score

Fifty-six (89%) patients with a Child-Pugh score A were compared to 7 (11%) patients with a Child-Pugh score B. Patients in the two groups had no differences in baseline characteristics except for: preoperative EV diagnosis (48 vs. 100%, *P*=0.009) and ligation rate (7 and 43%, *P*=0.005).

Concerning postoperative outcomes, intraoperative bleeding necessitating blood transfusion occurred more frequently in Child-Pugh score B patients (5 vs. 29%, *P*=0.032) (Table 3).

**Table 3:** Baseline characteristics and postoperative outcomes comparison between study groups based on Child-Pugh score.

	Child-Pugh A (*n*=56)	Child-Pugh B (*n*=7)	*P*
Baseline characteristics			
Age, years	56 (47.7–61)	58 (52–63)	0.677
Gender, M	22 (39)	4 (57)	0.366
Preoperative weight, kg	123.5 (110–140.7)	151 (128.5–159)	0.096
Preoperative BMI, kg/m^2^	44 (38.8–49.9)	51 (43.5–57.7)	0.130
Cardiovascular disease, *n*	35 (63)	5 (71)	0.644
T2D, *n*	39 (70)	7 (100)	0.098
OSAS, *n*	40 (71)	4 (57)	0.437
GERD, *n*	22 (39)	1 (14)	0.195
CKD, *n*	13 (23)	3 (43)	0.260
DVT, *n*	2 (4)	1 (14)	0.209
EV, *n*	27 (48)	7 (100)	0.009
Grade 1, *n*	20 (36)	5 (71)	0.069
Grade 2, *n*	5 (9)	2 (29)	0.119
EV ligation, *n*	4 (7)	3 (43)	0.005
Ascites, *n*	4 (7)	1 (14)	0.510
Splenomegaly, *n*	50 (89)	6 (86)	0.777
Cirrhosis etiology			
NASH, *n*	46 (82)	5 (71)	0.496
Viral, *n*	5 (9)	1 (14)	0.649
Other, *n*	9 (16)	1 (14)	0.903
TIPS, *n*	3 (5)	2 (29)	0.032
ASA score			
2, *n*	2 (4)	1 (14)	0.209
3, *n*	44 (79)	4 (57)	0.209
4, *n*	5 (9)	2 (29)	0.119
Bariatric procedure			
SG, *n*	41 (73)	6 (86)	0.474
RYGB, *n*	13 (23)	1 (14)	0.592
Other, *n*	2 (4)	0 (0)	0.611
Follow-up, months	42±21	34±10	0.327
Postoperative outcomes			
Decompensation			
Postoperative encephalopathy, *n*	1 (2)	0 (0)	0.721
General complications			
Postoperative renal failure, *n*	2 (4)	0 (0)	0.611
Dialysis, *n*	1 (2)	0 (0)	0.721
Portal vein thrombosis, *n*	3 (5)	0 (0)	0.530
Infection			
Sepsis, *n*	0 (0)	0 (0)	—
MBS complications			
Intraoperative bleeding^a^, *n*	3 (5)	2 (29)	0.032
Surgical postoperative complication, *n*	1 (2)	(0)	0.721
Leak, *n*	1 (2)	0 (0)	0.721
Mortality, *n*	1 (2)	0 (0)	0.721
Outcomes			
Postoperative weight, kg	97 (84–110)	129 (96.5–140)	0.077
Postoperative BMI^b^, kg/m^2^	34.6 (30.5–39.6)	42.2 (37–49.3)	0.036

Categorical variables are expressed as *n* (%). Continuous variables are reported as median (IQR).

*P*-values ≤0.05 are considered statistically significant.

a Intraoperative bleeding requiring blood transfusion.

b Postoperative BMI at last follow-up.

CKD, chronic kidney disease; DT2, type 2 diabetes; DVT, deep vein thrombosis; EV, esophageal varices; GERD, gastro-esophageal reflux disease; NASH, nonalcoholic steatohepatitis; OSAS, obstructive sleep apnea syndrome; RYGB, Roux-en-Y gastric bypass; SG, sleeve gastrectomy; TIPS, transjugular intrahepatic portosystemic shunt.

Univariate logistic regression showed that being in the Child-Pugh score A group was significantly associated with a reduced risk of intraoperative bleeding (OR: 0.73, 95% CI: 0.55–0.97, *P*=0.046). Multivariate logistic regression was then conducted including preoperative weight as a covariate showing a nonsignificant protective role of Child-Pugh score A towards intraoperative bleeding (OR: 0.11, 95% CI: 0.01–1.05, *P*=0.053). Then, we conducted multivariate analysis adjusted for EV grade and ligation, MELD>9, and previous finding of ascites, obtaining a nonsignificant protective factor of being Child-Pugh A score towards intraoperative bleeding (OR: 0.096; 95% CI: 0.004–1.33).

### MELD score

Concerning MELD score, 34 (54%) patients with a ≤9 MELD were compared to 29 (46%) with a >9 MELD (Table 4).

**Table 4:** Baseline characteristics and postoperative outcomes comparison between study groups based on MELD score.

	MELD≤9 (*n*=34)	MELD>9 (*n*=29)	*P*
Baseline characteristics			
Age, years	52 (46–60)	60 (51–62)	
Sex, M	13 (38)	13 (45)	0.457
Preoperative weight, kg	123 (109.2–138.4)	131 (113–145)	
Preoperative BMI, kg/m^2^	44 (38.9–49.5)	47.7 (39–54.2)	
Cardiovascular disease, *n*	20 (59)	20 (69)	0.520
T2D, *n*	23 (68)	23 (79)	0.475
OSAS, *n*	26 (76)	18 (62)	0.390
GERD, *n*	16 (47)	6 (21)	0.090
CKD, *n*	3 (9)	12 (41)	0.001
DVT, *n*	1 (3)	2 (7)	0.427
EV, *n*	16 (47)	19 (66)	0.142
grade 1, *n*	12 (35)	14 (48)	0.328
grade 2, *n*	4 (12)	3 (10)	0.929
EV ligation, *n*	1 (3)	6 (21)	0.020
Ascites, *n*	3 (9)	2 (7)	0.835
Splenomegaly, *n*	31 (91)	25 (86)	0.929
Cirrhosis etiology			
NASH, *n*	30 (88)	21 (72)	0.085
Viral, *n*	3 (9)	3 (10)	0.773
Other, *n*	4 (12)	6 (21)	0.280
TIPS, *n*	1 (3)	4 (14)	0.095
ASA score			
2, *n*	2 (6)	1 (3)	0.691
3, *n*	27 (79)	20 (69)	0.427
4, *n*	2 (6)	6 (21)	0.020
Bariatric procedure			
SG, *n*	20 (59)	26 (90)	0.017
RYGB, *n*	11 (32)	3 (10)	0.049
Other, *n*	2 (6)	0 (0)	0.199
Follow-up, months	29±15	30±23	0.837
Postoperative outcomes			
Decompensation			
Postoperative encephalopathy, *n*	0 (0)	1 (3)	0.260
General complications			
Postoperative renal failure, *n*	0 (0)	3 (10)	0.109
Dialysis, *n*	0 (0)	1 (3)	0.260
Portal vein thrombosis, *n*	0 (0)	3 (10)	0.109
Infection			
Sepsis, *n*	0 (0)	0 (0)	—
MBS complications			
Intraoperative bleeding^a^, *n*	1 (3)	4 (14)	0.095
Surgical postoperative complication, *n*	0 (0)	1 (3)	0.260
Leak, *n*	0 (0)	1 (3)	0.260
Mortality, *n*	0 (0)	1 (3)	0.260
Outcomes			
Postoperative weight, kg	97.7 (87.4–109)	99 (83.2–124.7)	
Postoperative BMI^b^, kg/m^2^	34.8 (30.6–38.1)	35.5 (31.2–42.3)	

Categorical variables are expressed as *n* (%). Continuous variables are reported as median (IQR).

*P*-values ≤0.05 are considered statistically significant.

a Intraoperative bleeding requiring blood transfusion.

b Postoperative BMI at last follow-up.

CKD, chronic kidney disease; DT2, type 2 diabetes; DVT, deep vein thrombosis; EV, esophageal varices; GERD, gastro-esophageal reflux disease; NASH, nonalcoholic steatohepatitis; OSAS, obstructive sleep apnea syndrome; RYGB, Roux-en-Y gastric bypass; SG, sleeve gastrectomy; TIPS, transjugular intrahepatic portosystemic shunt.

For baseline characteristics, patients with a >9 MELD score had an increased incidence of CKD (41 vs. 9%, *P*=0.001). Moreover, more patients in the >9 MELD score group had preoperative EV ligation (21 vs. 6%, *P*=0.02) and SG (90 vs. 59%, *P*=0.017) compared to the ≤9 MELD score group.

No significant differences were found regarding postoperative outcomes between the two groups. Univariate logistic regression was conducted for intraoperative bleeding necessitating for blood transfusion, portal vein thrombosis, and postoperative renal failure. In all cases, no significant increased risk was found for both patients groups so no multivariate logistic regression was conducted.

### Bariatric procedures

We conducted a subgroup analysis comparing patients who underwent SG (47, 77%) and RYGB (14, 23%). The results of baseline and postoperative outcomes comparisons are reported in Table 5.

**Table 5:** Baseline characteristics and postoperative outcomes comparison between study groups based on bariatric procedure.

	SG (*n*=47)	RYGB (*n*=14)	*P*
Baseline characteristics			
Age, years	56 (50–61)	57 (46–61)	0.612
Gender, M	21 (45)	4 (29)	0.282
Preoperative weight, kg	130 (111–143)	118 (104–130)	0.247
Preoperative BMI, kg/m^2^	45.6 (39.1–51.7)	43.6 (39–49.6)	0.577
Cardiovascular disease, *n*	31 (66)	8 (57)	0.547
T2D, *n*	35 (74)	10 (71)	0.856
OSAS, *n*	31 (66)	11 (79)	0.371
GERD, *n*	9 (19)	12 (86)	0.001
CKD, *n*	14 (30)	2 (14)	0.247
DVT, *n*	3 (6)	0 (0)	0.332
EV, *n*	26 (55)	6 (43)	0.412
grade 1, *n*	19 (40)	4 (29)	0.421
grade 2, *n*	5 (11)	2 (14)	0.707
EV ligation, *n*	5 (11)	2 (14)	0.707
Ascites, *n*	5 (11)	0 (0)	0.203
Splenomegaly, *n*	41 (87)	13 (93)	0.562
Cirrhosis etiology			
NASH, *n*	37 (79)	12 (86)	0.563
Viral, *n*	5 (11)	0 (0)	0.203
Other, *n*	8 (17)	2 (14)	0.808
TIPS, *n*	5 (11)	0 (0)	0.203
ASA score			
2, *n*	0 (0)	3 (21)	0.001
3, *n*	36 (77)	10 (71)	0.693
4, *n*	6 (13)	1 (7)	0.562
MELD score			
6–9, *n*	22 (47)	11 (79)	0.036
10–11, *n*	8 (17)	1 (7)	0.360
12–15, *n*	7 (15)	0 (0)	0.125
≥16, *n*	7 (15)	2 (14)	0.955
Child-Pugh			
A, *n*	41 (87)	13 (93)	0.562
B, *n*	6 (13)	1 (7)	
Follow-up, months	46±25	34±14	0.092
Postoperative outcomes			
Decompensation			
Postoperative encephalopathy, *n*	1 (2)	0 (0)	0.582
General complications			
Postoperative renal failure, *n*	2 (4)	0 (0)	0.433
Dialysis, *n*	1 (2)	0 (0)	0.582
Portal vein thrombosis, *n*	3 (6)	0 (0)	0.332
Infection			
Sepsis, *n*	0 (0)	0 (0)	—
MBS complications			
Intraoperative bleeding^a^, *n*	3 (6)	2 (14)	0.344
Surgical postoperative complication, *n*	1 (2)	0 (0)	0.582
Leak	1 (2)	0 (0)	0.582
Mortality, *n*	1 (2)	0 (0)	0.582
Outcomes			
Postoperative weight, kg	100 (85–110.6)	91.5 (78.3–101)	0.221
Postoperative BMI^b^, kg/m^2^	34.7 (30.8–40.9)	34.9 (32.5–36)	0.791

Categorical variables are expressed as *n* (%).

Continuous variables are reported as median (IQR).

*P*-values ≤0.05 are considered statistically significant.

a Intraoperative bleeding requiring blood transfusion.

b Postoperative BMI at last follow-up.

CKD, chronic kidney disease; DT2, type 2 diabetes; DVT, deep vein thrombosis; EV, esophageal varices; GERD, gastro-esophageal reflux disease; NASH, nonalcoholic steatohepatitis; OSAS, obstructive sleep apnea syndrome; RYGB, Roux-en-Y gastric bypass; SG, sleeve gastrectomy; TIPS, transjugular intrahepatic portosystemic shunt.

The two populations were comparable except for the fact that patients who received RYGB more frequently were assigned an ASA 2 (21 vs. 0%, *P*=0.001) and MELD ≤9 score (79 vs. 47%, *P*=0.036); moreover, patients undergoing RYGB presented more often preoperative GERD (86 vs. 19%, *P*=0.001). Preoperative weight and BMI were higher for patients who underwent SG as for postoperative weight and BMI, but this did not reach statistical significance. Concerning postoperative outcomes, no significant difference was highlighted between the two groups. For this reason, no univariate logistic regression was conducted.

## Discussion

The results of this multicenter, multinational study shed light on the feasibility and safety of MBS in a unique patient population characterized by morbid obesity and CSPH associated with cACLD. The study’s primary outcome, postoperative mortality, was 2% which is largely higher than the postoperative mortality of MBS that is around 0.1% as previously reported in a French administrative data analysis^14^. However, the present study demonstrates that postoperative mortality in this selected group of patients that have been considered not suitable candidates for MBS until recently, is lower than previously reported in older studies^15^. The lower mortality rate of ~2% contrasts with the findings of Mosko and Nguyen^4^ and Mumtaz *et al*.^16^, who reported a significantly higher mortality rate in the setting of cACLD. Although some recent studies^17,18^ on MBS performed in patients with cACLD highlighted similar mortality rates than the one reported in our research, in any case the authors focused on the concomitant presence of CSPH, which is generally associated with increased morbidity and mortality^7^.

There is general lack in the literature of research concerning perioperative evaluation and management as well as early and late postoperative outcomes in patients with liver cirrhosis undergoing nonhepatic surgery. However, there is general concordance about some fundamental points: 1) an extensive preoperative work-up to identify the severity of liver disfunction is mandatory; 2) every type of comorbidity, especially renal and cardiopulmonary, should be assessed preoperatively as it can furtherly affect postoperative outcomes; 3) the type of surgery and whether it is performed as a routine or emergency procedure are major determinants of outcomes; 4) preoperative risk stratification leads to a more precise prediction of outcomes as Child-Pugh score A and MELD <10 seem to be associated with a minimal increase in postoperative mortality compared to patients with more severe liver conditions; 5) strategies to reduce the risk of postoperative decompensation (i.e.: TIPS) should be adopted preoperatively^6,19,20^. However, there is no standardization in the above cited measures should be implemented and only few case series have been published assessing the utility of risk prediction tools and proposing recommendations to predict surgical outcomes and optimize cirrhotic patients conditions before surgery^21^.

One patient died in the present multicenter study after a staple line leak. Although the rate of staple line leak in SG has dramatically decreased in the last decade being as low as 0.2%^22^, morbidly obese individuals with cACLD associated with CSPH represent a very selected subgroup of patients and their functional reserve to face a life-threatening complication as the staple line leak may be diminished. The treatment in case of staple line leak after SG normally consists as a first step in nil per OS and broad spectrum antibiotics administration; when this is not sufficient and a peritoneal abscess is identified, radiologic-guided drainage or laparoscopic drainage are performed^23^. However, no specific recommendations concerning how to manage this complication in patients with liver cirrhosis exists. In recent years, the negative role of an inadequate nutritional status and of sarcopenia face to surgical complications has been highlighted^24,25^. Unfortunately, in the present study it was not possible to gather data concerning patients nutritional status and lean/fat body mass that would have been helpful to define the reasons responsible for the failure to rescue in this case.

The 2% postoperative mortality indicates a promising development and suggests an improvement in the safety of MBS. This aligns with the broader trend of decreased mortality rates in MBS over the years^14,26^. The inclusion criteria of this study required patients to be treated in advanced, tertiary referral centers for both MBS and liver transplantation. This selection of centers may have contributed to the improved outcomes by ensuring that patients receive specialized care for both their obesity and liver disease.

The study addresses the controversial issue of CSPH as a contraindication to BS. While the existing literature generally discourages MBS in CSPH, this study challenges that notion by demonstrating that, under specific circumstances, it can be performed with an acceptable level of safety. However, a few potentially life-threatening complications occurred in this series including portal thrombosis. The latter is a rare but potentially serious complication associated with MBS and almost exclusively with SG. Although the precise mechanism responsible for the increased risk of portal thrombosis in SG is still debated, in these selected patients, liver as prothrombotic factor must play a crucial role^27^ as in the present series this complication occurred in three out of 40 patients undergoing SG (7.5 of SG and 5% of the whole series). These findings should be taken into account when choosing which procedure should be performed in patients with CSPH. If patients are candidates for a liver transplantation and MBS is intended as a preparation for liver transplantation it should be considered that portal thrombosis may further complicate the access to transplantation and MBS should be better performed at the time of or after liver transplantation^28^. If no liver transplant is hypothesized, and the SG is chosen, then the anticoagulation should be adapted to this increased risk^29,30^. Furthermore, Pais *et al*.^31^ recently provided evidence that SG was an independent predictor, with advanced age, of the persistence of advanced fibrosis at long-term follow-up. We believe that it is crucial to be aware of the potential risk of portal thrombosis after SG as well as of its effects on liver fibrosis when choosing the bariatric procedure in the setting of cACLD. Portal venous system thrombosis after MBS in the general population of individuals with morbid obesity is a well-known entity. Its incidence is estimated to be 0.4% with SG and duodeno-jejunal bypass representing the procedures associated with the most increased incidence of this complication^32^; however, literature lacks data concerning the incidence of portal vein thrombosis in patients with cACLD and CSPH undergoing MBS. In this multicentric, international study, postoperative thromboprophylaxis was managed with low-molecular-weight heparin following national guidelines on this subject^33,34^. Nevertheless, also in this case there is no evidence supporting a particular thromboprophylaxis strategy for the specific population included in our research.

We also found five cases (8%) of intraoperative bleeding, which can be reliably linked to the increased portal pressure. This was significantly associated to Child-Pugh score B, which resulted to be a risk factor at univariate analysis; however, this was not confirmed at multivariate logistic regression. A few strategies exist to reduce the risk of bleeding including the TIPS, which was reported to be preoperatively performed in five (8%) patients in our series, and the use of staple line buttressing which has been shown to be effective in reducing the risk of bleeding^22,35,36^. Although we do not have data on the use of staple line buttressing in the present study, based on literature data, its use should be recommended in the setting of CSPH to reduce the risk of staple line bleeding.

The study adds to a limited body of evidence data suggesting that CSPH should not be an absolute contraindication to MBS. It aligns with a small number of prior studies that have explored this question, although it significantly expands the available evidence. However, clinicians and multidisciplinary teams should consider an individualized approach when assessing the suitability of MBS for patients with morbid obesity and CSPH. Factors such as the patient’s overall health and general conditions, nutritional status and presence sarcopenia, liver function, and the presence of other comorbidities should be carefully considered. In this specific setting, no consensus exists concerning well defined criteria to contraindicate MBS. Some of the included centers consider the intraoperative finding of ascites to be a contraindication to MBS as it represents a reflex of liver malfunction. However, other included centers reported MBS (both SG and RYGB) in patients with preoperative presence of ascites and no correlation with postoperative morbidity and mortality was highlighted although the small number of included patients can play a role in this. Ascites represents one of the most evident signs of decompensated liver disease and its finding in the intraoperative setting represented a contraindication for the realization of MBS for all the included centers. Furthermore, Precise criteria for selecting patients according to their degree of portal hypertension need to be defined in the future. In fact, CSPH was established by measuring HVPG in only one patient in the series. The expert centers involved in this study relied mainly on CT scans (whether or not patients had varices on endoscopy) to select good candidates for MBS, the extent of the perigastric localization of the porto-systemic shunt being a particularly important, but nonetheless rather subjective, factor. A precise classification based on imaging could be of interest to define surgical risk and better select surgical candidates in the future.

The involvement of advanced, specialized centers in the present study may account for the overall improved outcomes of MBS in the extreme conditions linked to CSPH. Therefore, patients in this category should be referred to centers with expertise in managing both obesity and chronic liver disease.

This study has several strength including the largest series reporting MBS in the setting of CSPH and the stringent inclusion criteria that allowed to gather a homogeneous study population to validate the concept that MBS can be safely done in this subgroup of morbidly obese individuals.

While this study provides valuable insights, it has some limitations including its retrospective nature, the potential selection bias, and the relatively small number of included cases. Furthermore, although the included patients respected the strict inclusion criteria set for this trial, a part of between patients heterogeneity persists as some of them had more severe liver disease, previous episodes of decompensation, and marks of a more severe portal hypertension (i.e.: grade II esophageal varices). For this reason, we conducted subgroup analysis comparing patients based on the Child-Pugh and MELD score to identify eventual differences in postoperative outcomes. The low number of the Child-Pugh score B group surely affected the results of statistics but it allowed to identify significant differences in variables distribution when discrepancy is important as in the case of intraoperative bleeding. Moreover, as the included centers were high-volume MBS tertiary centers with multidisciplinary teams including hepatobiliary surgeons, particular care should be taken in the generalization of the results to other hospital settings. Preoperative risk assessment and stratification are in any setting and the situation the fundamental premise to perform this surgery safely. Larger, prospective studies with longer follow-up periods are needed to confirm these results.

Finally, in the recent years the development of pharmacotherapeutic agents for the treatment of obesity has made significant advances with the introduction of semaglutide and tirzepatide (currently in phase 3 trials) and retatrutide (currently in phase 2 trial)^37^. The drugs proved to be highly effective in inducing weight loss and showed a safe profile and were associated with few side effects. Moreover, in vivo research showed that Glucagon-like protein-1 agonists also have a role in inducing liver fibrosis regression in patients with NASH^38^. However, although most of the individuals receiving these new drugs showed a substantial amount of weight loss, there is also an individual variability in the response to these drugs, mainly linked to the heterogeneity of obesity. This is why MBS will probably remain a valuable option in some selected cases also when these drugs will be largely available.

## Conclusion

In conclusion, this multicenter, multinational study contributes to our understanding of the feasibility and safety of MBS in individuals with morbid obesity and CSPH associated with cACLD. The results suggest that, when performed in specialized centers, BS in this patient population can have low postoperative mortality rates. This challenges the notion that CSPH should be an absolute contraindication to MBS. However, the decision to proceed with surgery in these cases should be individualized and made by a multidisciplinary team. Further research is warranted to confirm and expand upon these findings.

## Ethical approval

Due to the retrospective and anonymous nature of data, ethical approval was deemed not necessary.

## Source of funding

No funding supported this research.

## Author contribution

V.T.: writing original draft and data collection; O.M.G.: draft revision and data collection; J.K.H., M.L., F.R., A.-L.B., B.A., E.M., and H.A.: data collection; T.S.D., M.F., and H.T.: draft revision; C.B.: draft revision; T.P.: data collection and draft revision; A.C.: draft revision and statistical analysis; A.I.: draft revision, project management, and supervision.

## Conflicts of interest disclosure

The author declares no conflicts of interest.

## Research registration unique identifying number (UIN)

1. Name of the registry: ClinicalTrials.gov.
2. Unique identifying number or registration ID: NCT05653115.
3. Hyperlink to your specific registration (must be publicly accessible and will be checked): https://ichgcp.net/clinical-trials-registry/NCT05653115.

## Guarantor

Antonio Iannelli, MD, PhD.

## Data availability statement

Data is available through direct and motivated request to the authors.
